# Evaluation of Bacteria and Fungi DNA Abundance in Human Tissues

**DOI:** 10.3390/genes13020237

**Published:** 2022-01-27

**Authors:** Gabriela E. de Albuquerque, Bruno S. Moda, Marianna S. Serpa, Gabriela P. Branco, Alexandre Defelicibus, Isabella K. T. M. Takenaka, Maria G. de Amorim, Elizabeth C. Miola, Valquiria C. A. Martins, Katia L. Torres, Stephania M. Bezerra, Laura C. L. Claro, Adriane G. Pelosof, Claudia Z. Sztokfisz, Lais L. S. Abrantes, Felipe J. F. Coimbra, Luiz P. Kowalski, Fábio A. Alves, Stênio C. Zequi, Klas I. Udekwu, Israel T. Silva, Diana N. Nunes, Thais F. Bartelli, Emmanuel Dias-Neto

**Affiliations:** 1 Laboratory of Medical Genomics, A.C.Camargo Cancer Center, Sao Paulo 01508-010, SP, Brazil; gabi.albuquerque0602@gmail.com (G.E.d.A.); mariannaserpa@hotmail.com (M.S.S.); gbranco@gmail.com (G.P.B.); isabellakuniko@gmail.com (I.K.T.M.T.); maria.amorim@thermofisher.com (M.G.d.A.); liza.cris06@gmail.com (E.C.M.); dnoronha@accamargo.org.br (D.N.N.); 2 Laboratory of Computational Biology and Bioinformatics, A.C.Camargo Cancer Center, Sao Paulo 01508-010, SP, Brazil; brunomoda@gmail.com (B.S.M.); alexandre.defelicibus@accamargo.org.br (A.D.); itojal@accamargo.org.br (I.T.S.); 3 Department of Education and Research, Fundação Centro de Controle de Oncologia do Estado do Amazonas, Manaus 69040-010, AM, Brazil; alvesvalquiria@yahoo.com.br (V.C.A.M.); katialuztorres@hotmail.com (K.L.T.); 4 Department of Pathology, A.C.Camargo Cancer Center, Sao Paulo 01509-001, SP, Brazil; stephania.bezerra@accamargo.org.br (S.M.B.); lauralopez502@gmail.com (L.C.L.C.); 5 Rede D’Or São Luiz S/A, Sao Paulo 04321-130, SP, Brazil; 6 Santa Casa de Misericórdia de São Paulo, Sao Paulo 01221-010, SP, Brazil; 7 Endoscopy, A.C.Camargo Cancer Center, Sao Paulo 01509-001, SP, Brazil; adriane.pelosof@accamargo.org.br (A.G.P.); claudia.sztokfisz@accamargo.org.br (C.Z.S.); 8 International Research Center, A.C.Camargo Cancer Center, Sao Paulo 01508-010, SP, Brazil; lais.senda@accamargo.org.br; 9 Director Department of Abdominal Surgery, Head Upper GI Oncology Reference Center, A.C.Camargo Cancer Center, Sao Paulo 01509-001, SP, Brazil; felipe.coimbra@accamargo.org.br; 10 Department of Head and Neck Surgery and Otorhinolaryngology, A.C.Camargo Cancer Center, Sao Paulo 01509-001, SP, Brazil; lpkowalski@accamargo.org.br; 11 Department of Head and Neck Surgery, University of Sao Paulo Medical School, Sao Paulo 01246-903, SP, Brazil; 12 Department of Stomatology, A.C.Camargo Cancer Center, Sao Paulo 01509-001, SP, Brazil; falves@accamargo.org.br; 13 Department of Urology, A.C.Camargo Cancer Center, Sao Paulo 01509-001, SP, Brazil; stenio.zequi@accamargo.org.br; 14 National Institute for Science and Technology in Oncogenomics and Therapeutic Innovation, A.C.Camargo Cancer Center, São Paulo 01509-001, SP, Brazil; 15 Department of Aquatic Sciences and Assessment, Swedish University of Agriculture, P.O. Box 7050, 75007 Uppsala, Sweden; klas.udekwu@slu.se; 16 Department of Medical Sciences, Gastroenterology/Hepatology, Uppsala University Akademiska Sjukhuset, Ingång 40, 75185 Uppsala, Sweden; 17 Laboratório de Neurociências Alzira Denise Hertzog Silva, Instituto de Psiquiatria, Hospital das Clínicas, Faculdade de Medicina, Universidade de São Paulo, Sao Paulo 05403-010, SP, Brazil

**Keywords:** microbiome, mycobiome, metagenomics, 16S, qPCR, shotgun

## Abstract

Whereas targeted and shotgun sequencing approaches are both powerful in allowing the study of tissue-associated microbiota, the human: microorganism abundance ratios in tissues of interest will ultimately determine the most suitable sequencing approach. In addition, it is possible that the knowledge of the relative abundance of bacteria and fungi during a treatment course or in pathological conditions can be relevant in many medical conditions. Here, we present a qPCR-targeted approach to determine the absolute and relative amounts of bacteria and fungi and demonstrate their relative DNA abundance in nine different human tissue types for a total of 87 samples. In these tissues, fungi genomes are more abundant in stool and skin samples but have much lower levels in other tissues. Bacteria genomes prevail in stool, skin, oral swabs, saliva, and gastric fluids. These findings were confirmed by shotgun sequencing for stool and gastric fluids. This approach may contribute to a more comprehensive view of the human microbiota in targeted studies for assessing the abundance levels of microorganisms during disease treatment/progression and to indicate the most informative methods for studying microbial composition (shotgun versus targeted sequencing) for various samples types.

## 1. Introduction

The human microbiota consists of trillions of microorganisms, including viruses, archaea, bacteria, fungi, protists, and other eukaryotes that may impact human health. The transition to an unbalanced, dysbiotic state needs to be explored, as it may be either the cause or the result of numerous pathological states. The reduction in sequencing costs and the continuous development of high throughput bioinformatics pipelines have contributed immensely not only to study human-associated microorganisms, but also to a deeper view of the microbiota associated with different niches, including plants [1,2], soils [3,4], urban environment [5,6], hospitals [7,8], and even the international space-station [9]. Notably, advances in the study of human-associated microbiota allowed the investigation of numerous health and disease aspects, ranging from the impact of C-section versus vaginal delivery on the microbiota of newborns [10,11], to neurodegenerative diseases [12,13] and the microbiota impact in various cancers [14,15], including treatment response [16].

In general, the most desirable and informative protocols for microbiota studies involve total DNA extraction, followed by DNA fragmentation, library construction, and sequencing, which is denominated shotgun metagenomics. This approach, depending on the efficacy of the DNA/RNA extraction, would allow the capture of all nucleic acids (total nucleic acids—TNA) available and provide a less skewed view of the microbiota [17]. From a comparative standpoint, the human genome is much larger (10–10,000×) than those of viruses, bacteria, fungi, or parasites, thus confounding comparison between studies. Furthermore, depending on the sample source, the human cell fraction will significantly outnumber the microbiota, when the host DNA can easily dominate the resultant TNA pool. This confounding issue may affect the proper inference of the microbiota and impede its effective characterization. Particularly, when the ratio of human to non-human DNA is less favorable, shotgun sequencing would be severely impacted, as most sequencing reads (>99.9%) would originate from the host [18]. In this sense, several strategies have recently emerged as alternatives to provide enrichment of microbial versus host DNA. Most of these methods rely on eliminating host cells prior to DNA extraction or require an intact, high-quality molecular weight DNA, which may depend on the sample source. Furthermore, the potential bias when determining microbial community profiles has not been thoroughly explored and their efficacy is variable and still debatable [19].

Analyses of the tissue-associated microbiota remain limited to cultivation protocols or the study of targeted regions of the genome, such as 16S rRNA for bacteria or ITS for fungi [20,21,22,23]. While the former is impacted by the lack of protocols that would allow the unbiased growth of all microorganisms in the sample, as to reflect their sample-specific abundance, the latter imposes limitations such as representation bias and lower phylogenetic resolution [24,25]. These biases have affected both sample-source and experimental approach selection (shotgun or targeted) for microbiota investigations. Consequently, most publications have focused on the study of the gut microbiota, where the bacteria: human ratios are very favorable for microbiome studies. Of particular importance, the tissue microbiota, which has been shown to impact immunomodulation and chemotherapy response in oncology [26], would benefit from characterization via more comprehensive (shotgun) approaches. Body tissues contain important and unique microbiota, whose function and interest are being unraveled [27,28], and would likewise benefit. In accordance with this, were non-human: human DNA ratios quantified, appropriate samples for targeted or shotgun metagenomic studies could readily be determined, allowing a cost-effective, and more comprehensive or less biased microbiota evaluation. In addition to complementing and strengthening the potential of target and shotgun sequencing for microbiome composition analysis, determining the ratio of non-human DNA in a sample can be informative of the fluctuations in the abundance of the microbiota in several biological conditions, including therapeutic interventions such as chemotherapy and radiotherapy in cancer patients [16,29].

Here, we propose a unifying approach to determine the relative and absolute abundance of fungi and bacteria in different human sample types. This combines the comparison of targeted gene amplification and shotgun sequencing, both assessing the relative proportions of each DNA source for nine tissue types.

## 2. Materials and Methods

### 2.1. Samples and DNA Extraction Methods

A total of 87 samples were included, representing nine different sample types: oral mucosa swabs (*n* = 10), non-stimulated saliva (*n* = 10), gastric fluids collected during endoscopy (*n* = 16), stool (*n* = 5), biopsies of salivary glands (*n* = 6), gastric biopsies (*n* = 10), biopsies of rectal tumors (*n* = 10), tissue fragments from penile tumors (*n* = 10), and skin swabs (*n* = 10). Some tissue specimens were selected as they were simple to obtain and would represent different tissue types (such as oral and skin swabs, and saliva) or when they would allow the characterization of body niches that are often explored in oncology, such as biopsies, tissue samples, and gastric fluids. Subjects enrolled were volunteers from ongoing studies in our group, to evaluate the microbiota of cancer patients and controls, or members of our research group.

Gastric fluids and gastric biopsies were collected during endoscopy for diagnostic purposes at the A.C.Camargo Cancer Center and stored at −20 °C). DNA from oral and skin swabs were extracted with DNeasy PowerSoil Kit (Qiagen, Hilden, NW, Germany); saliva and gastric fluids with phenol:chloroform:isoamyl alcohol 25:24:1 (Merck, Damstadt, HE, Germany); salivary gland, rectum, penis, and stool DNA was obtained with E.Z.N.A.^®^ Bacterial DNA Kit (Omega Bio-Tek, Norcross, GA, USA), and gastric biopsies DNA was extracted with AllPrep DNA/RNA Mini Kit (Qiagen). DNA samples were quantified with Qubit™ dsDNA HS Assay Kit (ThermoFisher, Waltham, MA, USA) and qPCR amplified.

### 2.2. Primer Pairs and qPCR DNA Amplification

Bacteria- and fungi-derived DNA, were respectively evaluated by DNA amplification of a short fragment of the V1 region of the 16S rRNA (V1_F: 5′-AGAGTTTGATCMTGGCTCAG-3′ and V1_R: 5′-TTACTCACCCGTICGCCRCT-3′) or a portion of the 5.8S rRNA region (5.8S_F: 5′-CARCAAYGGATCTCTTGG-3′ and 5.8S_R: 5′-TGTGCGTTCAAAGATTCGAT-3′). To evaluate the human DNA levels, used to normalize human:fungi:bacteria ratios, we used *ACTB* primers (ACTB_F: 5′-CCATCTACGAGGGGTATGC-3′ and ACTB_R: 5′-GGTGAGGATCTTCATGAGGTA-3′) that amplify two targets in the human genome (chrms 5 and chrms 7), generating amplicons of similar lengths (88 and 92 nt). The qPCR mix for *ACTB* and 16S-V1 genes consisted of 0.2 μM of each primer, 5 μL of Fast SYBR Green 2× (ThermoFisher) and 3 μL of the eluted DNA in various concentrations, in a final volume of 10 μL. Cycling conditions consisted of an initial denaturation (95 °C for 20 s) followed by 40 cycles at 95 °C for 3 s and 60 °C for 30 s. For 5.8S gene fragment, the qPCR mix included 0.2 μM of each primer, 5 μL of the GoTaq Master Mix 2× (Promega, Madison, WIS, USA) and 4.2 μL of eluted DNA in a final volume of 10 μL. Cycling conditions for qPCR were initial denaturation at 95 °C for 3 min, followed by 40 cycles at 95 °C for 15 s and 58 °C for 30 s. All experiments were conducted in duplicates and qPCR was performed in a 7500 Real-Time PCR System (ThermoFisher).

### 2.3. Standard Curves

**Figure 1:**
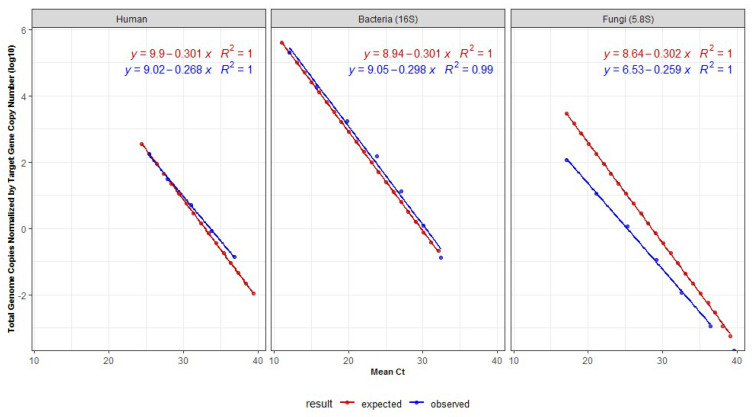
Standard DNA dilution curves for human, bacteria (V1-16S) and fungi (5.8S) qPCR, showing expected (red) and observed (blue) Ct-values considering the genome size and target copy number of the bacteria and fungi present in the mock community. Calculations do not consider the variable ploidy observed in many fungi species. Values used for plotting are shown in Table 1. In the equation, y denotes the genome copy number while x denotes the mean Ct value.

For absolute quantitative analysis by qPCR, we utilized commercially available human genomic DNA (Promega—G1471) to generate a standard curve derived from human amplicons. The ZymoBIOMICS™ Microbial Community DNA Standard (Zymo Research—D6305/D6306,) composed of eight bacteria and two fungi species (*Pseudomonas aeruginosa, Escherichia coli, Salmonella enterica, Lactobacillus fermentum, Enterococcus faecalis, Staphylococcus aureus, Listeria monocytogenes, Bacillus subtilis, Saccharomyces cerevisiae, Cryptococcus neoformans*), was used to prepare standard curves for bacteria and fungi, starting with a DNA mass of 5 ng. The number of bacteria, fungi, or human genomes were calculated per ng of DNA according to the formula: number of copies = mass of DNA used (ng) × 6.022 × 10^23^/average genome size × (1 × 10^9^) × 650 (average weight of a base pair)—divided by the median number of copies of the target genes. Mean Cts corresponding to each dilution point were used for plotting the standard curves. Mean genome size and target copy number were considered for the bacteria and fungi present in the mock community. A median of six copies of 16S rRNA for bacteria, 102.5 copies of rRNA for the haploid fungi (https://rrndb.umms.med.umich.edu, accessed on 13 April 2020), and four for the human calculations (two copies of the two loci amplified by the ACTB primers in a diploid human genome). The genome sizes considered were 6.4 Gb for the human diploid genome, 3.58 Mb for bacteria, and 15.6 Mb for fungi haploid genomes (Figure 1, Table 1). These values represent the median of the genome sizes for the components of the mock according to the National Center for Biotechnology (NCBI) database at the time of analysis (https://www.ncbi.nlm.nih.gov/genome/, accessed on 13 April 2020).

For calculating the absolute genome copy number for the specimens used here, additional standard curves were created considering the genome sizes and target gene copy number of an increased number of bacteria and fungi, not limited to the ones present at the mock community. For the construction of these standard curves and calculations, the average genome sizes were considered as 6.4 Gb for humans (human diploid genome), 12.3 Mb (fungi haploid genome), and 4.15 Mb (bacteria). The values for bacteria and fungi were respectively calculated based on the median length of 25,923 bacteria and 177 fungi genomes available at https://www.ncbi.nlm.nih.gov/genome/, accessed on 2 December 2021. Only complete genome sequences in the database were considered. The number of copies of the target genes was five for the bacterial 16S rRNA (based on a total of 20,255 full bacteria genomes from the rrnDB database (as of Oct. 2021-https://rrndb.umms.med.umich.edu, accessed on 2 December 2021); and 82 copies of the rRNA for fungi (based on a total of 91 fungal taxa, as described by Lofgren et al., 2019). Calculations of the genome copies (Log10) were obtained as follow: (i) human genome copies = 9.02 − (0.268 × mean Ct value); (ii) bacteria genome copies = 9.06 − (0.298 × mean Ct value), and (iii) fungi genome copies 6.73 − (0.259 × mean Ct value). Ploidy was not considered for the overall calculations in fungi and may have impacted calculations in real-life samples. Absolute genome copies were calculated for bacteria and fungi according to the Cts obtained for each sample after comparing them to the standard curves. Standard curves correlating the mean Cts with the total genome copies/ng of DNA were constructed. Each standard curve is given as: (i) expected Cts—considered according to the lowest Ct-value, under the assumption that a difference of one Ct would correspond to a 2× difference in the dilution of the template DNA mass and (ii) observed Cts—real values observed after qPCR amplification for the same theoretical dilutions.

### 2.4. Shotgun Sequencing

As another metric to evaluate bacteria: human and fungi: human ratios, and to determine the accuracy of our calculations, we compared qPCR ratios to shotgun metagenomic sequencing data of 10 samples of gastric fluids and two stool samples. For this, we used 100 ng of DNA template for library preparation for each sample (Nextera DNA Flex kit, Illumina, San Diego, CA, USA), followed by sequencing on the NextSeq 500 platform (Illumina). The results were analyzed by Kraken2 [30], and the number of reads for bacteria, fungi and human were used to calculate the relative frequency of each group, and to compare with qPCR data.

### 2.5. Statistical Analysis

Possible significant differences between the genome copy number of the different samples were assessed by the Mann–Whitney test. For the correlation analyses, we used Spearman’s correlation. For all statistical tests, a level of 5% significance was considered (*p* < 0.05). Statistical analysis and graphs were generated with the appropriate packages using R Studio.

## 3. Results

### 3.1. Standard Curves Correlating Genome Copies and Cts

Standard curves were plotted, allowing to correlate the approximate absolute number of genome copies for bacteria and fungi according to the respective Cts achieved in qPCR amplification (Table 1). As shown in Figure 1, all standard curves showed very high correlations (*R²* equal to 0.99 or 1), despite some discrepancies observed for fungi.

### 3.2. Impacts of Varying Bacteria:Human and Fungi:Human DNA-Ratios over Amplification/Quantification Capabilities

**Figure 2:**
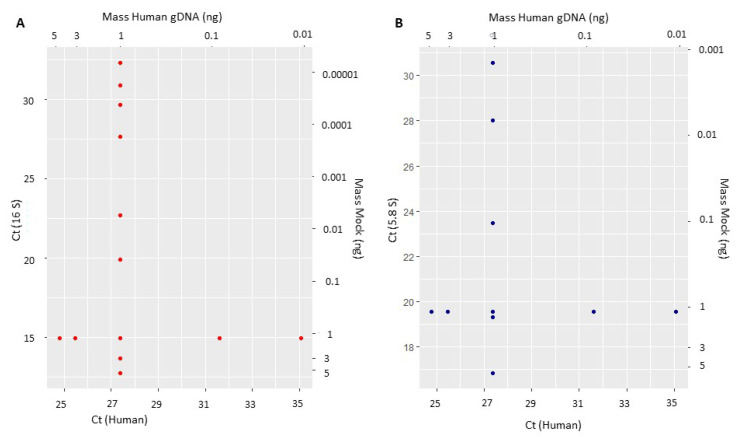
Effects of varying DNA mass over amplification capabilities of bacteria (**A**) and fungi (**B**), as compared to human DNA amounts. Horizontal axes indicate the Ct value for human DNA and vertical axes indicate Cts for bacteria (16S) or fungi (5.8S). Each dot represents the mean of a triplicate experiment.

In order to simulate real-life analysis of samples that present significant variations in the ratios of target microorganisms and host-derived DNA, we evaluated the Ct fluctuations in face of different human: bacteria and fungi: human DNA ratios and their possible impact on the quantification of these microorganisms. When using a fixed amount of human DNA (1 ng) and up to 500× variation (5 ng to 0.01 ng) of the mock DNAs from bacteria or fungi, the Ct values for the human ACTB gene remained within the expected range of variation, suggesting no inhibition or off-target amplification. The same was observed when the amount of mock DNA was fixed at 1ng, and the human DNA varied 500× (0.01 ng to 5 ng), with no significant impact on either the bacteria 16S-, or the fungi 5.8S-detection (Figure 2).

### 3.3. Genome Copies in Different Sample-Types

**Figure 3:**
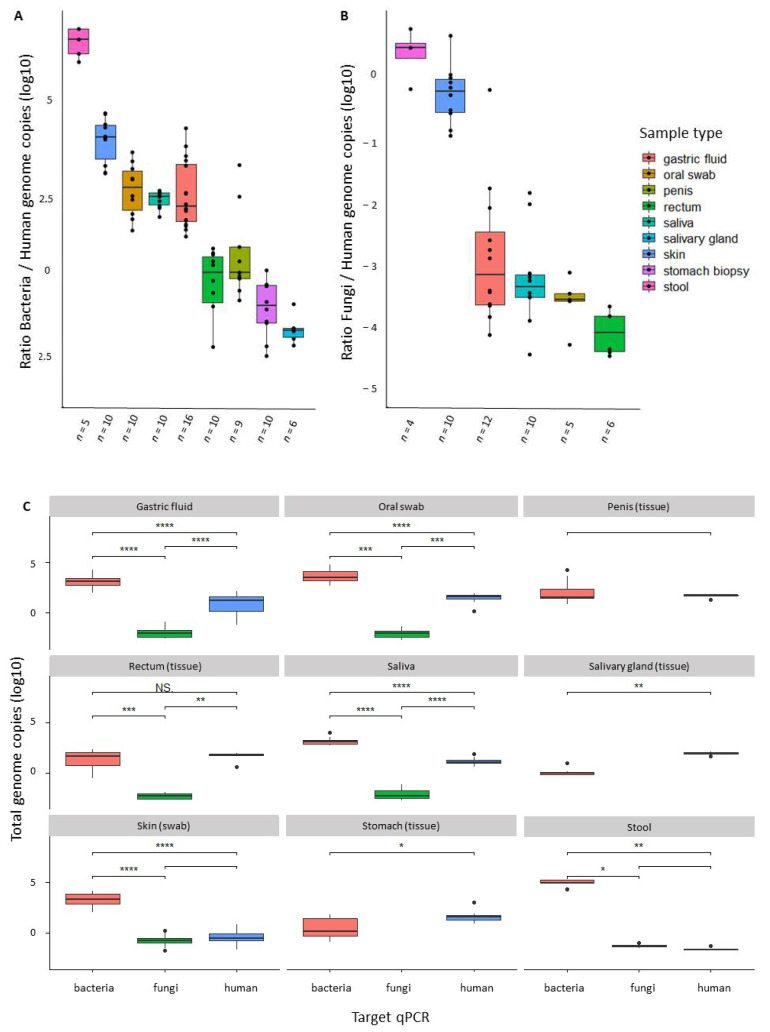
Genome-ratios for bacteria:human (**A**) and fungi:human (**B**) and total genome copies for bacteria (red), fungi (green) and human (blue) (**C**) in different human sample types. Mann–Whitney test * *p* < 0.05; ** *p* < 0.01; *** *p* < 0.001; **** *p* < 0.0001 significant.

After running the standard curves for different amounts of DNA and showing no significant variations of background human DNA to affect the expected Cts, we were able to determine the approximate number of bacteria or fungi genome copies for nine different types of human-derived samples. Figure 3A shows, as expected, that stool samples have the highest bacteria: human ratios—making this a very good source for metagenome shotgun sequencing, as published by many different groups. Our data also show that skin and oral swabs, saliva, and gastric fluids also contain more bacteria genomes than human genomes and, thus, should provide informative microbiota data using this same sequencing approach, despite the much larger length of the human genome. By contrast, some tissues contain similarly high levels of human and bacterial genomes—as with rectal and penile biopsies—suggesting that metagenomics shotgun sequencing could still be informative, but human DNA will largely dominate due to its length.

After comparing the approximate number of human and bacteria genomes in all samples, we observed that biopsies from salivary gland and stomach contained much less bacteria genomes as compared to human genomes (Figure 3C). Rectal and penile biopsies appear to have similar amounts of human and bacterial genome copies, whereas the other sample types have significantly more bacteria, stool being the sample with most bacterial DNA as expected. From our limited set of samples, only stool and skin samples seem to have more significant fungi: human genome-ratios, and good variation was seen for gastric fluids and saliva. Oral swabs, saliva, and gastric fluids have similar proportions of genomes from each type of organism, which makes sense as the gastric microbiota is heavily influenced by the large amounts of microorganisms present in the saliva (Figure 3C).

The quantitative analysis of fungi by qPCR showed stool and skin to be the samples with more fungi genome copies, with a reduction in saliva, oral swabs, rectal biopsies, all presenting similar amounts of fungi genomes. Interestingly, very small amounts of human genome were found in the skin and stool, as compared with the other tissues and samples evaluated here as DNA sources (Figure 3B,C).

### 3.4. Shotgun Analysis

As shotgun sequencing is less biased, it was used to compare the resulting data with our qPCR data, to analyze the relative abundance of bacteria, fungi, and human-derived DNA in 16 samples of gastric fluids and two stool samples. Our data has shown human-derived reads varying from 0.5 and 67% in the gastric fluids, and to be ≤0.1% in the stool. As expected, we observed a smaller contribution of fungi-DNA, as compared to bacteria for both sample types. This low frequency of fungi was true for all samples, ranging from 0.03 to 0.6% of the total reads in gastric fluids, and 0.1% in the two stool samples.

**Figure 4:**
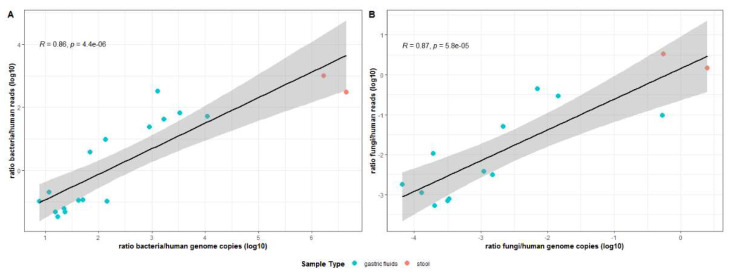
Correlation between the ratios of bacteria:human reads observed by shotgun sequencing data and the relative bacteria:human genome copies calculated by qPCR (**A**). The same is shown for fungi (**B**). Each circle represents a distinct sample. For statistical analysis, Spearman’s correlation was used, and the closer it is to 1, the greater the correlation between the variables. The shaded area represents the confidence interval. Correlations were considered as statistically significant when *p* < 0.05.

Our shotgun sequencing results were also compared in terms of bacteria: human and fungi: human genome ratios (obtained from qPCR analysis of the same samples). As can be seen in Figure 4, very good correlations were obtained between qPCR data and the ratios of bacteria: human reads (Figure 4A) and fungi: human reads (Figure 4B), from shotgun sequencing analysis. This reinforces the validity of the herein presented qPCR approach, as well as the supporting calculations that allow the amounts of the genomes of these organisms to be determined.

## 4. Discussion

Most human microbiota studies are focused on the composition and diversity of the microbiota and do not consider that the amounts of bacteria or the human: bacteria ratios could be informative. Additionally, very few authors have focused on the mycobiome composition, not to mention the different fungi colonization enrichment in sample types and disease settings, and possible impacts of this biomass in regulating or having any role in biological processes. Moreover, the quantitative variation of the microbiota may not only result from disease processes but from the use of medicines, pre-and pro-biotics, and drugs such as alcohol and tobacco, as well as levels of mucosa immunity and other situations. Here we observed that only stool and skin samples had more fungi genomes, but still much lower than bacteria, as compared to the other sample types. This observation should, however, be viewed with caution, as very few specimens were evaluated for some tissue types. On the other hand, fungi such as *Candida albicans* are known as important members of the human gastrointestinal tract and may also be opportunistic pathogens [31]. In this sense, it seems feasible that in some pathologies and for different tissue types, variations in the number of microorganisms may have a physiological role and may suggest the impact of certain drugs or therapies on the microbiota, their role in drug metabolization, or even serve as a proxy of immune surveillance status where microorganisms appear to influence immune regulation [32,33,34].

Other applications of our approach are more technical and include the use of this tool to effectively indicate the best sequencing approach for determining microbiota composition, and how many reads one can expect from a shotgun metagenomics study. In this sense, our results demonstrated that gastric tissues had a significantly higher number of human genomes per bacterial or fungi genomes as compared to the gastric fluids from the same patients, indicating that a shotgun approach for gastric microbiome studies should be more efficient if based on gastric fluids. From our data (Figure 3) it is also clear that samples from skin, oral swabs, and gastric fluids have amounts of non-human DNA that would allow a direct shotgun sequencing approach, with no need to first amplify the target genes. Indeed, this was confirmed from our shotgun sequencing of gastric fluids. As seen here and for other studies, the quantification of fungi may be less precise due to ploidy variation, which may occur according to species composition and developmental stage [35,36], as well as the higher complexity of the fungal cell wall, which is usually more resistant to lysis [37], and would also affect fungi quantification and analysis when shotgun approaches are used. Overall, it seems that our approach is reliable, as our amplifications showed no detectable fluctuations when facing 500× excess of human-host DNA or 500× excess of mock microbiota template DNA, and it is also capable to provide a precise microbiota quantification, as seen from the expected/observed number of reads for bacteria or fungi as well as the amount of human-derived DNA in shotgun metagenomics. A good demonstration of this is the evaluation of the microbiota of gastric fluids by shotgun metagenomics, as predicted by our quantitative analysis, which shows that >50% of samples have more bacteria-derived DNA than host-DNA. Our data also indicates samples for which the shotgun approach would be inadequate, when lesser amounts of bacteria- or fungi-derived DNA are present, precluding the microbiota evaluation with shotgun sequencing.

The application of this method for medical/biological situations of interest is envisioned. An example may include the monitoring of bacteria biomass after antibiotic treatment and the resolution of the sceptic shock [38], the observation of variations in the abundance of protective or opportunistic microorganisms that may impact surgery, radiotherapy, and chemotherapy, including side-effects such as infections of surgical sites, mucositis, and even treatment efficacy [39,40,41]. In this regard, the use of specific microorganisms to protect the intestinal epithelium has been suggested to manage collateral damage related to radiotherapy [42,43]. Our approach could thus be applied to investigate a number of treatment-related scenarios including the impact of ‘protective’ bacteria inoculation in pre-radiotherapy versus post-radiotherapy stages, treatment efficacy, and to assess later development of mucositis in different grades. We envisage the widespread application of this simple tool to rapidly assess bacterial levels in diverse pathophysiological and other clinically relevant scenarios of metagenomics interest, not limited to human samples.

## 5. Conclusions

In this study, we proposed a method for estimating the relative and absolute abundance of fungi and bacteria in human samples. We observed that the ratio of human per bacteria or fungi DNA is variable depending on the sample collection method (tissue, swab, body fluids) and source (tissues such as skin, rectum, stomach, and penis). Additionally, although bacteria outnumber fungi in most samples, we show that these microorganisms can be more abundant in a few sample types (stool and skin), indicating their importance in the microbiome of different body sites. This approach provides a valuable resource for the improvement of metagenomic studies, enabling a more comprehensive view of the human microbiota in targeted studies and a quantitative assessment of the microbiota in several clinical scenarios.

## Figures and Tables

**Table 1:** Calculated correlations between DNA mass, mean Cts and number of copies of the genomes and the target genes for the mock DNA and human DNA.

Organism Group	DNA Mass in qPCR (ng)	Mean Ct	Genome Lenght (Mb)	Number of Genome Copies	Number of Genomes Normalized by Copies of the Target	Log10 of the Number Genomes
Mock-bacteria	4.800000	12.1	3.6	1,242,233.8	207,038.9	5.3
	0.432432	15.8	3.6	111,913.0	18,652.16	4.3
	0.038958	19.8	3.6	10,082.2	1680.37	3.2
	0.003510	23.8	3.6	908.3	151.39	2.2
	0.000316	27.1	3.6	81.8	13.64	1.1
	0.000028	30.0	3.6	7.4	1.23	0.1
	0.000003	32.4	3.6	0.7	0.11	−1.0
Human	5.000000	25.4	64,000	723.9	180.96	2.3
	0.833333	27.8	64,000	120.6	30.16	1.5
	0.138889	31.1	64,000	20.1	5.03	0.7
	0.023148	33.8	64,000	3.4	0.84	−0.1
	0.003858	36.9	64,000	0.6	0.14	−0.9
Mock-fungi	0.200000	17.1	15.6	11,875.1	115.9	2.1
	0.020000	21.1	15.6	1187.5	11.6	1.1
	0.002000	25.3	15.6	118.8	1.2	0.06
	0.000200	29.2	15.6	11.9	0.1	−0.9
	0.000020	32.6	15.6	1.2	0.01	−1.9
	0.000002	36.4	15.6	0.1	0.001	−2.9

Notes: 1—The total mass of the mock contained 96% of bacteria DNA (12% of each of the eight species) and 4% from fungi DNA (2% of each one of the two species). 2—Calculations have considered the average number of copies of the amplification targets in the respective genomes used in this mock and the diploid human genome (four for human DNA, six for bacteria, and 102.5 for fungi).

## Data Availability

The data that support the findings of this study are available on request from the corresponding authors (T.F.B.; E.D.-N.).

## References

[1] Khan Z., Doty S.L. (2009). Characterization of bacterial endophytes of sweet potato plants. Plant Soil.

[2] Hardoim P.R., van Overbeek L.S., van Elsas J.D. (2008). Properties of bacterial endophytes and their proposed role in plant growth. Trends Microbiol..

[3] Baldrian P., Kolarik M., Štursová M., Kopecky J., Valášková V., Větrovský T., Žifčáková L., Šnajdr J., Rídl J., Vlček Č. (2012). Active and total microbial communities in forest soil are largely different and highly stratified during decomposition. ISME J..

[4] Auffret M.D., Karhu K., Khachane A., Dungait J., Fraser F., Hopkins D., Wookey P., Singh B., Freitag T.E., Hartley I.P. (2016). The Role of Microbial Community Composition in Controlling Soil Respiration Responses to Temperature. PLoS ONE.

[5] Mason C., Afshinnekoo E., Ahsannudin S., Ghedin E., Read T., Fraser C., Dudley J., Hernandez M., Bowler C., Stolovitzky G. (2016). The Metagenomics and Metadesign of the Subways and Urban Biomes (MetaSUB) International Consortium inaugural meeting report. Microbiome.

[6] Danko D., Bezdan D., Afshin E.E., Ahsanuddin S., Bhattacharya C., Butler D.J., Chng K.R., Donnellan D., Hecht J., Jackson K. (2021). A global metagenomic map of urban microbiomes and antimicrobial resistance. Cell.

[7] Lax S., Sangwan N., Smith D., Larsen P., Handley K.M., Richardson M., Guyton K., Krezalek M., Shogan B.D., Defazio J. (2017). Bacterial colonization and succession in a newly opened hospital. Sci. Transl. Med..

[8] Chng K.R., Li C., Bertrand D., Ng A.H.Q., Kwah J.S., Low H.M., Tong C., Natrajan M., Zhang M.H., Xu L. (2020). Cartography of opportunistic pathogens and antibiotic resistance genes in a tertiary hospital environment. Nat. Med..

[9] Checinska Sielaff A., Urbaniak C., Mohan G.B.M., Stepanov V.G., Tran Q., Wood J.M., Minich J., McDonald D., Mayer T., Knight R. (2019). Characterization of the total and viable bacterial and fungal communities associated with the International Space Station surfaces. Microbiome.

[10] Biasucci G., Rubini M., Riboni S., Morelli L., Bessi E., Retetangos C. (2010). Mode of delivery affects the bacterial community in the newborn gut. Early Hum. Dev..

[11] Mueller N.T., Whyatt R., Hoepner L., Oberfield S., Dominguez-Bello M.G., Widen E.M., Hassoun A., Perera F., Rundle A. (2015). Prenatal exposure to antibiotics, cesarean section and risk of childhood obesity. Int. J. Obes..

[12] Poole S., Singhrao S.K., Kesavalu L., Curtis M.A., Crean S. (2013). Determining the Presence of Periodontopathic Virulence Factors in Short-Term Postmortem Alzheimer’s Disease Brain Tissue. J. Alzheimer’s Dis..

[13] Scheperjans F., Aho V., Pereira P.A.B., Koskinen K., Paulin L., Pekkonen E., Haapaniemi E., Kaakkola S., Eerola-Rautio J., Pohja M. (2015). Gut microbiota are related to Parkinson’s disease and clinical phenotype. Mov. Disord..

[14] Thomas A.M., Jesus E.C., Lopes A., Aguiar S.J., Begnami M.D., Rocha R.M., Carpinetti P.A., Camargo A.A., Hoffmann C., Freitas H.C. (2016). Tissue-Associated Bacterial Alterations in Rectal Carcinoma Patients Revealed by 16S rRNA Community Profiling. Front. Cell. Infect. Microbiol..

[15] Metagenomic Analysis of Colorectal Cancer Datasets Identifies Cross-Cohort Microbial Diagnostic Signatures and a Link with Choline Degradation|Nature Medicine. https://www.nature.com/articles/s41591-019-0405-7.

[16] Lehouritis P., Cummins J., Stanton M., Murphy C.T., McCarthy F., Reid G., Urbaniak C., Byrne W.L., Tangney M. (2015). Local bacteria affect the efficacy of chemotherapeutic drugs. Sci. Rep..

[17] Quince C., Walker A.W., Simpson J.T., Loman N.J., Segata N. (2017). Shotgun metagenomics, from sampling to analysis. Nat. Biotechnol..

[18] Marotz C.A., Sanders J.G., Zuniga C., Zaramela L.S., Knight R., Zengler K. (2018). Improving saliva shotgun metagenomics by chemical host DNA depletion. Microbiome.

[19] Heravi F.S., Zakrzewski M., Vickery K., Hu H. (2020). Host DNA depletion efficiency of microbiome DNA enrichment methods in infected tissue samples. J. Microbiol. Methods.

[20] Gophna U., Sommerfeld K., Gophna S., Doolittle W.F., van Zanten S.J.O.V. (2006). Differences between Tissue-Associated Intestinal Microfloras of Patients with Crohn’s Disease and Ulcerative Colitis. J. Clin. Microbiol..

[21] Thomas A.M., Gleber-Netto F.O., Fernandes G.R., Amorim M., Barbosa L.F., Francisco A.L.N., de Andrade A.G., Setubal J.C., Kowalski L.P., Nunes D.N. (2014). Alcohol and tobacco consumption affects bacterial richness in oral cavity mucosa biofilms. BMC Microbiol..

[22] Esposito S., De Simone G., Gioia R., Noviello S., Pagliara D., Campitiello N., Rubino C., Pardo D.L., Boccia G., DE Caro F. (2017). Deep tissue biopsy vs. superficial swab culture, including microbial loading determination, in the microbiological assessment of Skin and Soft Tissue Infections (SSTIs). J. Chemother..

[23] Aykut B., Pushalkar S., Chen R., Li Q., Abengozar R., Kim J.I., Shadaloey S.A., Wu D., Preiss P., Verma N. (2019). The fungal mycobiome promotes pancreatic oncogenesis via activation of MBL. Nature.

[24] Donachie S.P., Foster J.S., Brown M.V. (2007). Culture clash: Challenging the dogma of microbial diversity. ISME J..

[25] Campanaro S., Treu L., Kougias P.G., Zhu X., Angelidaki I. (2018). Taxonomy of anaerobic digestion microbiome reveals biases associated with the applied high throughput sequencing strategies. Sci. Rep..

[26] Anfossi S., Calin G.A. (2020). Gut microbiota: A new player in regulating immune- and chemo-therapy efficacy. Cancer Drug Resist..

[27] Chng K.R., Chan S.H., Ng A.H.Q., Li C., Jusakul A., Bertrand D., Wilm A., Choo S.P., Tan D.M.Y., Lim K.H. (2016). Tissue Microbiome Profiling Identifies an Enrichment of Specific Enteric Bacteria in Opisthorchis viverrini Associated Cholangiocarcinoma. eBioMedicine.

[28] Poore G.D., Kopylova E., Zhu Q., Carpenter C., Fraraccio S., Wandro S., Kosciolek T., Janssen S., Metcalf J., Song S.J. (2020). Microbiome analyses of blood and tissues suggest cancer diagnostic approach. Nature.

[29] Kim Y.S., Kim J., Park S.-J. (2015). High-throughput 16S rRNA gene sequencing reveals alterations of mouse intestinal microbiota after radiotherapy. Anaerobe.

[30] Wood D.E., Lu J., Langmead B. (2019). Improved metagenomic analysis with Kraken 2. Genome Biol..

[31] Pérez J.C. (2021). Fungi of the human gut microbiota: Roles and significance. Int. J. Med. Microbiol..

[32] Geller L.T., Barzily-Rokni M., Danino T., Jonas O.H., Shental N., Nejman D., Gavert N., Zwang Y., Cooper Z.A., Shee K. (2017). Potential role of intratumor bacteria in mediating tumor resistance to the chemotherapeutic drug gemcitabine. Science.

[33] Yuan L., Zhang S., Li H., Yang F., Mushtaq N., Ullah S., Shi Y., An C., Xu J. (2018). The influence of gut microbiota dysbiosis to the efficacy of 5-Fluorouracil treatment on colorectal cancer. Biomed. Pharmacother..

[34] Guthrie L., Gupta S., Daily J., Kelly L. (2017). Human microbiome signatures of differential colorectal cancer drug metabolism. NPJ Biofilms Microbiomes.

[35] Todd R.T., Forche A., Selmecki A. (2017). Ploidy Variation in Fungi: Polyploidy, Aneuploidy, and Genome Evolution. Microbiol. Spectr..

[36] Wertheimer N.B., Stone N., Berman J. (2016). Ploidy dynamics and evolvability in fungi. Philos. Trans. R. Soc. B Biol. Sci..

[37] Fredricks D.N., Smith C., Meier A. (2005). Comparison of Six DNA Extraction Methods for Recovery of Fungal DNA as Assessed by Quantitative PCR. J. Clin. Microbiol..

[38] Jimenez M.F., Marshall J.C. (2001). Source control in the management of sepsis. Intensiv. Care Med..

[39] Wenzel R.P. (2019). Surgical site infections and the microbiome: An updated perspective. Infect. Control Hosp. Epidemiol..

[40] Subramaniam N., Muthukrishnan A. (2019). Oral mucositis and microbial colonization in oral cancer patients undergoing radiotherapy and chemotherapy: A prospective analysis in a tertiary care dental hospital. J. Investig. Clin. Dent..

[41] Irfan M., Delgado R.Z.R., Frias-Lopez J. (2020). The Oral Microbiome and Cancer. Front. Immunol..

[42] He J.-Y., Wang W.-Z., Qi H.-Z., Ma Y., He S.-Y. (2018). Use of recombinant Lactobacillus sakei for the prevention and treatment of radiation-induced enteritis. Med. Hypotheses.

[43] Riehl T.E., Alvarado D., Ee X., Zuckerman A., Foster L., Kapoor V., Thotala D., Ciorba M.A., Stenson W.F. (2018). Lactobacillus rhamnosus GG protects the intestinal epithelium from radiation injury through release of lipoteichoic acid, macrophage activation and the migration of mesenchymal stem cells. Gut.

